# Corollary discharge inhibition of wind-sensitive cercal giant interneurons in the singing field cricket

**DOI:** 10.1152/jn.00520.2014

**Published:** 2014-10-15

**Authors:** Stefan Schöneich, Berthold Hedwig

**Affiliations:** Department of Zoology, University of Cambridge, Cambridge, United Kingdom

**Keywords:** *Gryllus bimaculatus*, corollary discharge, stridulation, cercal escape pathway, postsynaptic inhibition

## Abstract

Crickets carry wind-sensitive mechanoreceptors on their cerci, which, in response to the airflow produced by approaching predators, triggers escape reactions via ascending giant interneurons (GIs). Males also activate their cercal system by air currents generated due to the wing movements underlying sound production. Singing males still respond to external wind stimulation, but are not startled by the self-generated airflow. To investigate how the nervous system discriminates sensory responses to self-generated and external airflow, we intracellularly recorded wind-sensitive afferents and ventral GIs of the cercal escape pathway in fictively singing crickets, a situation lacking any self-stimulation. GI spiking was reduced whenever cercal wind stimulation coincided with singing motor activity. The axonal terminals of cercal afferents showed no indication of presynaptic inhibition during singing. In two ventral GIs, however, a corollary discharge inhibition occurred strictly in phase with the singing motor pattern. Paired intracellular recordings revealed that this inhibition was not mediated by the activity of the previously identified corollary discharge interneuron (CDI) that rhythmically inhibits the auditory pathway during singing. Cercal wind stimulation, however, reduced the spike activity of this CDI by postsynaptic inhibition. Our study reveals how precisely timed corollary discharge inhibition of ventral GIs can prevent self-generated airflow from triggering inadvertent escape responses in singing crickets. The results indicate that the responsiveness of the auditory and wind-sensitive pathway is modulated by distinct CDIs in singing crickets and that the corollary discharge inhibition in the auditory pathway can be attenuated by cercal wind stimulation.

discrimination of self-generated (reafferent) and external (exafferent) signals that occur simultaneously in the same sensory pathway during active behavior is a fundamental challenge for all animals (Cullen 2004; Sperry 1950; von Holst and Mittelstaedt 1950). Using corollary discharge or an “efference copy” of the motor commands to modulate or cancel the self-generated signals during sensory processing is an effective neuronal mechanism to prevent inappropriate behavioral responses and also desensitization of the sensory pathway due to reafferent stimulation (Crapse and Sommer 2008; Poulet and Hedwig 2007). Corollary discharge mechanisms are found in animals with very different levels of neural complexity (Bell 1989; Davis et al. 1973; Reznik et al. 2014; Robertson and Moulins 1981; Yang et al. 2008; Zaretsky and Rowell 1979), and there is accumulating evidence that dysfunction of corollary discharge circuits in the human brain may cause clinical psychopathologies like schizophrenia (Feinberg and Guazzelli 1999; Pynn and DeSouza 2013).

The field cricket with its rather simple nervous system and stereotypic singing behavior is an excellent model system to study the neurophysiological and cellular mechanisms of corollary discharge processing in sensory pathways (Poulet 2005). To attract conspecific females, the male crickets sing by rhythmically opening and closing the raised forewings and thereby produce one syllable (short sound pulse) during each closing movement. In the calling song of the Mediterranean field cricket *Gryllus bimaculatus*, chirps comprising 4 ± 1 syllables are perseveringly repeated at a rate of 2–3 Hz, and the syllable repetition rate within the chirps is in a range of 25–30 Hz (Ferreira and Ferguson 2002; Schöneich and Hedwig 2012). During singing, when the hearing system of the cricket is exposed to its own loud chirps, a single pair of corollary discharge interneurons (CDI) provides rhythmic pre- and postsynaptic inhibition of auditory afferents and first-order interneurons, respectively (Poulet and Hedwig 2006). By reducing the neuronal responses to the self-generated sound pulses, the corollary discharge effectively protects the cricket's auditory pathway from self-induced desensitization (Poulet and Hedwig 2002).

Besides sound, however, the periodic wing movements of a singing cricket produce also notable airflow around the animal's body (Kämper and Dambach 1985). These self-generated air currents seem not to play a significant role in communication (Pollack et al. 1998), but do have particle velocities well above the threshold of the cercal mechanoreceptors to activate giant interneurons (GIs) of the wind-sensitive escape pathway in a resting cricket (Kämper and Dambach 1981; Kumagai et al. 1998; Magal et al. 2006). Although spiking of the cercal GIs is known to initiate escape behavior (Boyan and Ball 1990; Gnatzy and Hustert 1989; Gras and Hörner 1992; Hörner 1992; Kohstall-Schnell and Gras 1994; Tauber and Camhi 1995), male crickets usually sing continuously for several hours, while additional external wind stimulation is still effective to elicit transient silencing and escape reactions (Dambach et al. 1983; Hedwig 2000; Lewkiewicz and Zuk 2004).

To investigate how the central nervous system discriminates between self-generated and external wind stimuli, here we intracellularly recorded the activity of individual wind-sensitive cercal afferents and GIs in the terminal ganglion of fictively singing crickets. If neurons of the cercal escape pathway receive rhythmic inhibition during fictive singing, a situation without any self-induced sensory feedback, it must be the result of a corollary discharge from the central pattern generator for singing.

## MATERIALS AND METHODS

### 

#### Animals.

All experiments were performed with male, mature Mediterranean field crickets (*Gryllus bimaculatus* DeGeer). The animals were selected 1–2 wk after their final molt from the cricket colony maintained under crowded conditions at 28°C on a 12:12-h light-dark cycle in the Department of Zoology (University of Cambridge, UK). The experiments were carried out at room temperature (20–25°C) and complied with the principles of Laboratory Animal Care.

#### Preparation and fictive singing.

**Fig. 1.**
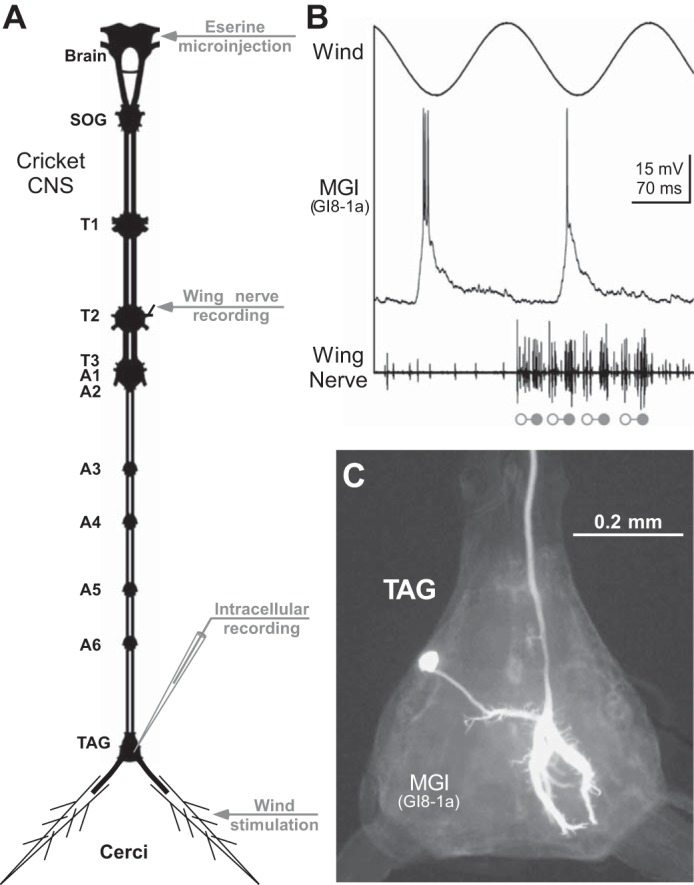
Experimental design. *A*: diagram of the cricket central nervous system (CNS) indicates the location of the mesothoracic wing-nerve (T2-N3A) recording, eserine injection into the brain, intracellular recording in the terminal abdominal ganglion (TAG), and the cercal wind stimulation. *B*: synaptic and spike response of a wind-sensitive cercal giant interneuron (*middle* trace) to sinusoidal cercal wind stimulation (*top* trace) during fictive singing. Open and solid circles indicate opener and closer motoneuron spike bursts, respectively, of a four-syllable chirp in the wing nerve recording (*bottom* trace). *C*: photomicrograph of the Lucifer Yellow labeled interneuron in the TAG allows subsequent anatomical identification as the median giant interneuron (MGI), also known as GI8-1a, according the classification by Jacobs and Murphey (1987).

After legs and wings were removed, the crickets were opened by a dorsal longitudinal incision and pinned out ventral side down onto a plasticine-covered platform. The ganglia of the ventral nerve cord were exposed for electrophysiological recording and continually perfused with Ringer's saline (concentrations in mmol/l: NaCl 140, KCl 10, CaCl_2_ 7, NaHCO_3_ 8, MgCl_2_ 1, *N*-trismethyl-2-aminoethanesulfonic acid 5, d-trehalose dihydrate 4; pH 7.4). The peripheral nerves of all three thoracic and the terminal abdominal ganglion (TAG) were cut, except for the sensory cercal nerves. The head of the cricket was waxed to a moveable metal support, and a small window was cut in the forehead cuticle to gain access to the brain. Fictive singing was elicited by pressure-injection (Pneumatic PicoPump PV820; WPI, Sarasota, FL) of the acetylcholine esterase inhibitor eserine (10^−2^ M in saline; Sigma-Aldrich, St. Louis, MO) into the ventral protocerebrum using a blunt glass-microcapillary (Schöneich and Hedwig 2011; Wenzel et al. 1998). From the mesothoracic nerve 3A (Fig. 1*A*), the motor pattern of fictive singing was recorded with a double-hook electrode (Schöneich et al. 2011) and amplified with a differential AC-amplifier (model 1700; A-M Systems, Sequim, WA). In the following, we refer to this nerve branch as wing nerve, as it contains axons of wing-opener and wing-closer motoneurons (cf., Schöneich and Hedwig 2012).

#### Intracellular recording and staining.

Cercal afferents were recorded close to their axonal terminals in the neuropile of the terminal ganglion, and the CDI and wind-sensitive interneurons were recorded in their dendritic branches in the mesothoracic (Poulet and Hedwig 2006) and terminal ganglion (Jacobs and Murphey 1987), respectively. For intracellular recording, the mesothoracic ganglion and/or terminal ganglion were stabilized between a silver ring and a subjacent silver platform with an embedded optic fiber for bright-field illumination. Sharp microelectrodes were pulled (DMZ-Universal Puller, Zeitz-Instruments, Martinsried, Germany) from borosilicate glass capillaries (GC100F-10, Harvard Apparatus, Kent, UK). Tips were filled with 4% Lucifer Yellow (Sigma-Aldrich, St. Louis, MO), and the shaft was backfilled with 1 mol/l lithium chloride. Microelectrodes had final resistances of 90–130 MΩ. Intracellularly recorded signals were amplified using a DC-amplifier with current injection facility (BA-01X, NPI, Tamm, Germany). For anatomical identification, recorded neurons were subsequently labeled by iontophoretic injection of Lucifer Yellow. After the experiment, the respective ganglia were dissected, fixed in 4% paraformaldehyde (2 h at room temperature), dehydrated in an ascending ethanol series and finally cleared in methyl salicylate.

#### Cercal wind stimulation.

Sinusoidal wind stimuli to the cerci were delivered via a pipette tip of 1 mm inner diameter, which was positioned about 50 mm behind the animal, pointing toward the cercus. Wind signals were generated by a mini-shaker unit (Mini-Shaker and Amplifier Type 27184810, Brüel and Kjaer Naerum, Denmark) that displaced a rubber diaphragm of an air chamber when driven by the sinusoidal voltage signal of a function generator (model TG-101, Thandar Electronics, Huntingdon, UK). The sinusoidal movements of the diaphragm caused air pressure changes in the chamber, which were transmitted to the pipette tip via a short pneumatic tube (cf., Meyer and Hedwig 1995). During fictive singing, crickets could not produce any air currents, as the wings were removed and the thoracic ganglia were de-efferented. Stimulus frequencies ranged between 1 and 100 Hz to cover the response range of wind-sensitive cercal hairs (cf., Kämper and Dambach 1981). Stimulus amplitude was individually adjusted during each experiment to sufficiently stimulate the cercal system without triggering silencing reactions that would stop the singing motor activity (Fig. 1*B*).

#### Data sampling and analysis.

All electrophysiological recordings were monitored with an analog oscilloscope (Tektronix 5440) and simultaneously digitized with 40-kHz sampling rate per channel (Micro1401 mk II; CED, Cambridge, UK) for storage on a PC hard drive. Off-line data analysis was performed with Spike2 (CED, Cambridge, UK) and Neurolab (Knepper and Hedwig 1997) software. For quantitative data analysis, we predominantly used the singing motor activity in the wing nerve as temporal reference. If not stated otherwise, time = 0 ms in the figures corresponds to the first wing-opener motoneuron spike of the chirp in the mesothoracic wing nerve N3A. Before signal averaging, the extracellular recording traces of the wing nerve were full-wave rectified to prevent cancelation of biphasic spike signals (cf., Schöneich and Hedwig 2010). As the number of syllables per chirp can vary between and change during pharmacologically induced fictive singing activity, we analyzed for signal averages and poststimulus time histograms only chirps with the same number of syllables. Mean values are given with standard deviations (mean ± SD; *n*: number of analyzed events; *N*: number of animals) for all normally distributed data. For data that failed testing for Gaussian distribution (D'Agostino and Pearson omnibus normality test), the median value is given, and statistical differences between datasets were tested with Mann-Whitney *U*-test (Prism 5.0, GraphPad, La Jolla, CA). Stained neurons (Fig. 1*C*) were visually inspected and photographed using a digital single-lense reflex camera (Canon EOS 350D) mounted on a fluorescence microscope (Axiophot, Zeiss, Germany). The ascending wind-sensitive interneurons were identified by their unique morphologies described by Jacobs and Murphey (1987). The mesothoracic CDI was identified by its distinctive anatomical and physiological characteristics described by Poulet and Hedwig (2006).

## RESULTS

The dendrites of the cercal GIs are located within the cercal glomerulus of the terminal ganglion (Jacobs and Murphey 1987), where they receive monosynaptic excitatory inputs from wind-sensitive cercal afferents (Matsumoto and Murphey 1977; Shepherd et al. 1988). Among the different types of cercal mechanoreceptors (Gnatzy and Hustert 1989), the long and slender filiform hairs are most sensitive to wind stimulation (Landolfa and Miller 1995; Miller et al. 2011; Shimozawa and Kanou 1984) and activate ascending GIs to initiate directed predator avoidance reactions (Gras and Hörner 1992; Hörner 1992; Kanou et al. 1999; Matsumoto and Murphey 1977; Tobias and Murphey 1979).

To investigate how the nervous system deals with self-generated and external wind stimuli during singing, we intracellularly recorded wind-sensitive cercal afferents and their postsynaptic ascending GIs in the terminal ganglion of fictively singing crickets. In such a de-efferented preparation with only the sensory cercal nerve unsevered, a corollary discharge inhibition would be indicated by reduced responsiveness of wind-sensitive cercal interneurons whenever external wind stimulation coincides with the centrally generated motor activity. A response reduction in the first-order interneurons, however, can be caused either by direct postsynaptic inhibition at the dendritic branches of the interneuron or by presynaptic inhibition of the afferent terminals, or may reflect the concurrent impact of pre- and postsynaptic inhibition.

### 

#### Activity of wind-sensitive cercal afferents during fictive singing.

**Fig. 2.**
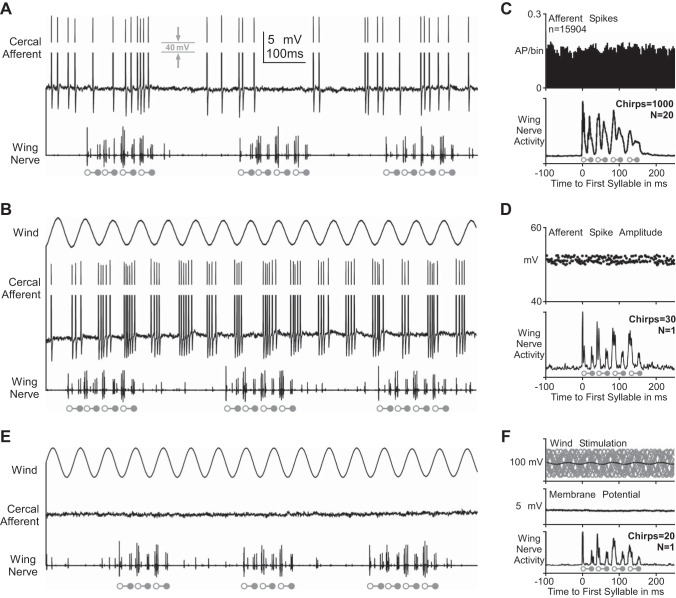
Intracellular recordings of wind-sensitive cercal afferents and wing motor nerve activity during fictive singing. Open and solid circles indicate the opener and closer motoneuron spike burst, respectively, of each syllable (*bottom* traces). Spontaneous (*A*) and wind-evoked (*B*) afferent spike activity, and afferent membrane potential after transection of the cercal nerve (*E*) are shown. *C*: histogram shows no correlation between afferent spike activity and the motor pattern of fictive singing. AP, action potential. *D*: the singing motor pattern did not influence the spike amplitude of wind-sensitive afferents close to their presynaptic terminals (average of 30 chirps from one representative experiment; *n* = 186 afferent spikes). *F*: average of afferent membrane potential in the terminal ganglion after cercal nerve transection shows no modulation with the singing rhythm (average of 20 chirps; cercal wind stimulation: stimulus overlay as gray lines, and stimulus average as black line).

To test for presynaptic corollary discharge inhibition, we intracellularly recorded cercal afferents close to their axonal terminals in the terminal ganglion of 20 fictively singing crickets. Evidence for presynaptic inhibition should, therefore, be reflected in the presence of primary afferent depolarization, which shunts the membrane resistance and systematically reduces the afferent spike amplitudes (Levy 1977; Nishi et al. 1974). Without external wind stimulation, the sensory afferents of filiform hairs irregularly generated action potentials with a mean spike frequency of 10–50 Hz (Fig. 2*A*). When exposed to a 10- to 20-Hz sinusoidal wind-stimulus, they responded to each cycle with a burst of 3–6 action potentials (100- to 250-Hz mean spike frequency within bursts), while no spike activity occurred in the anti-phase of the rhythmic stimulation (Fig. 2*B*). In none of the afferent recordings (*N* = 20 animals) did we find any indication for rhythmic spike activity modulation in phase with the fictive singing motor activity (Fig. 2*C*). Neither spontaneous nor wind-elicited spike occurrence was affected by the generation of the singing motor pattern (Fig. 2, *A*–*C*). We found also no systematic reduction of afferent action potential amplitudes in any phase of the fictive singing rhythm (Fig. 2, *A*, *B*, and *D*). In the most stable afferent recording, the median values of spike amplitudes during chirp and during chirp interval were both 51.1 mV, and the amplitude distributions within the two groups also did not differ significantly (Mann-Whitney *U* = 4,295, *n*_chirp_ = 95, *n*_interval_ = 91, *P* = 0.94, two-tailed; 30 chirps analyzed). We also found no significant spike amplitude difference between chirps and chirp intervals in any of the other afferent recordings (Mann-Whitney *U*-test, *P* > 0.5, two-tailed). Furthermore, we did not observe any rhythmic depolarization of the membrane potential in the axonal terminals during spontaneous (Fig. 2*A*) or wind-evoked spiking (Fig. 2*C*) or after eliminating any spike activity by surgical transection of the sensory cercal nerve (Fig. 2, *E* and *F*). As we found no evidence for primary afferent depolarization during fictive singing, our data suggest that the central generation of singing motor activity may not entail presynaptic corollary discharge inhibition of the wind-sensitive afferents in the cercal pathway.

#### Rhythmic inhibition of ventral GIs during singing.

We further recorded in the terminal ganglion the dendritic membrane potential of the two ventral GIs, GI8-1a (*N* = 6) and GI8-1b (*N* = 1), to analyze their synaptic and spike activity during fictive singing.

**Fig. 3.**
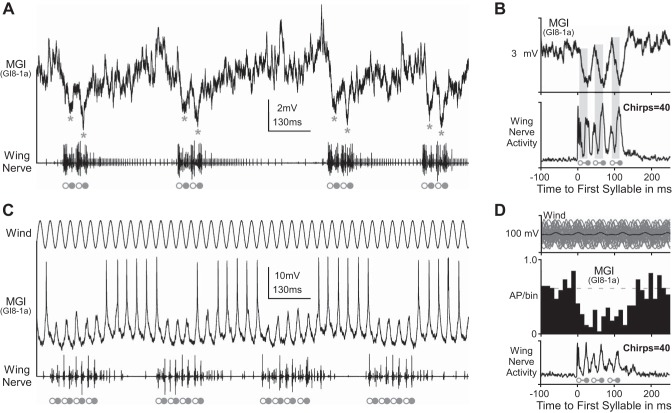
Corollary discharge inhibition (CDI) of the median giant fiber (GI8-1a) during fictive singing. Open and solid circles indicate the opener and closer motoneuron activity, respectively, in the wing nerve (*bottom* traces). *A*: intracellular recording from the MGI dendrite shows postsynaptic inhibition (marked by asterisks) during each syllable of fictive singing. *B*: the inhibition starts strictly during the opener phase of each syllable and reaches its maximum in the subsequent closer phase (signal average of 40 chirps; gray bars indicate the time window of inhibitory inputs). *C*: the spike response of the MGI to cercal wind stimulation is reduced by CDI during fictive chirps. *D*: histogram (*n* = 464 APs) shows that the reduced responsiveness of GI8-1a to cercal wind stimulation is coupled to the motor pattern of fictive singing (average of 40 chirps).

Intracellular recordings of GI8-1a, which is also known as the median GI (MGI), revealed inhibitory postsynaptic potentials (IPSPs) occurring strictly in phase with the syllable pattern (Fig. 3, *A* and *B*). Due to spontaneous afferent activity, the MGI is permanently driven by a barrage of background excitatory postsynaptic potentials with amplitudes mostly below the spiking threshold. Therefore, the inhibitory inputs that accompanied fictive singing (asterisks in Fig. 3*A*) were often masked and not very obvious in the recording traces. Averaging the dendritic membrane potential fluctuations over several chirps, however, demonstrated a rhythmic corollary discharge inhibition in phase with the syllable rhythm of fictive singing for every intracellular MGI recording (*N* = 6 animals). The average compound IPSPs in the dendrite reached −3.5 ± 2.3 mV (*N* = 6). The inhibition started 6.3 ± 2.2 ms (*N* = 6) after the onset of the wing-opener motoneuron burst in the wing nerve and reached its maximum 3.7 ± 1.8 ms (*N* = 6) after the first spike of the wing-closer motoneuron burst (see Fig. 3*B*). The spike response of the MGI was always significantly reduced whenever the cercal wind stimulation coincided with the fictive singing motor activity (Mann-Whitney *U*-test: *P* < 0.001 each; *N* = 6, *n* = 25 chirp cycles analyzed for each animal). Depending on intensity, frequency and direction of the external wind stimulation and its precise timing to the syllable rhythm of the fictive singing pattern, the overall reduction of spike response during the chirp phases ranged in the different experiments between 40% and 95% (*N* = 6). In response to sinusoidal wind stimulation at the syllable frequency of about 30 Hz, for example, the MGI reliably produced 1–2 action potentials per stimulation cycle during the chirp intervals, whereas the spike response was largely suppressed during the chirps (Fig. 3, *C* and *D*).

**Fig. 4.**
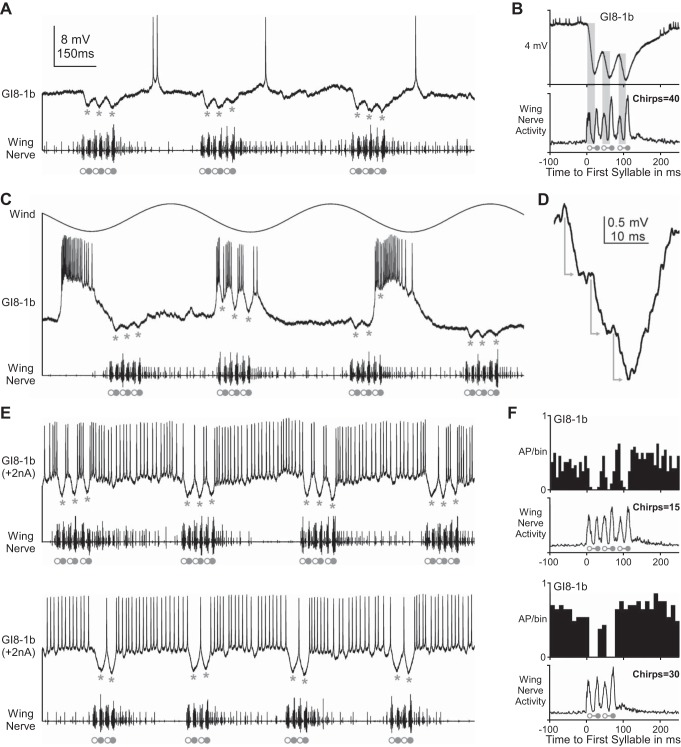
CDI of the cercal giant interneuron GI8-1b during fictive singing. Open and solid circles indicate the opener and closer motoneuron spike burst, respectively, in the wing nerve (*bottom* traces). Spontaneous (*A*), wind-evoked (*C*), and current injection driven (*E* and *F*) spike activity of GI8-1b is rhythmically reduced by inhibitory postsynaptic potentials (IPSPs; marked by asterisks) during fictive singing. *B*: GI8-1b receives inhibitory inputs (highlighted by gray bars) during the opener phase of each syllable; the inhibition reaches its maximum at the beginning of the subsequent closer phase (signal average of 40 chirps). *D*: for each syllable of the chirp, a large compound IPSP results from summation of 3–4 individual IPSPs (indicated by gray arrows). *E*: tonic spike activity of GI8-1b, induced by 2-nA depolarizing current injection, was interrupted by CDI in phase with the syllable pattern of the singing motor activity. *F*: spike timing histograms for 3- and 2-syllable chirps (15 chips: *n* = 394 spikes and 30 chirps: *n* = 634 spikes, respectively) show the reduced spike activity of GI8-1b occurring in phase with the fictive syllable pattern.

We recorded similar postsynaptic inhibition in phase with the syllable rhythm of fictive singing in the dendrite of the ventral GI, GI8-1b (Fig. 4, *A*–*C*). The inhibition started 5.5 ± 2.5 ms (*n* = 100 syllables) after the onset of the wing-opener motoneuron burst in the wing nerve and reached its maximum 1.6 ± 1.8 ms (*n* = 100 syllables) before the start of the wing-closer motoneuron activity (Fig. 4*B*). For each syllable, the inhibitory input consisted of 3–4 small IPSPs (Fig. 4*D*) that occurred at a frequency of 190 ± 22 Hz and summated to an overall compound IPSPs of −3.1 ± 0.7 mV (*n* = 100 syllables). The spike activity of GI8-1b in response to cercal wind stimulation was reduced whenever the stimulus coincided with the fictive singing motor activity (Fig. 4*C*). Furthermore, driving spike activity in GI8-1b by constantly depolarizing its dendrite with +2 nA current injection demonstrated that the inhibition of spike activity in this giant fiber is tightly coupled to the syllable rhythm of the singing motor pattern (Fig. 4, *E* and *F*).

In summary, our recordings demonstrate that corollary discharge from the singing pattern generator rhythmically inhibits the two prominent ventral giant fibers GI8-1a (MGI) and GI8-1b in phase with the motor output that controls the wing-opener movements and thereby reduces their responsiveness to cercal wind stimulation during the singing activity.

#### Interaction between the wind-sensitive pathway and the CDI for auditory processing.

**Fig. 5.**
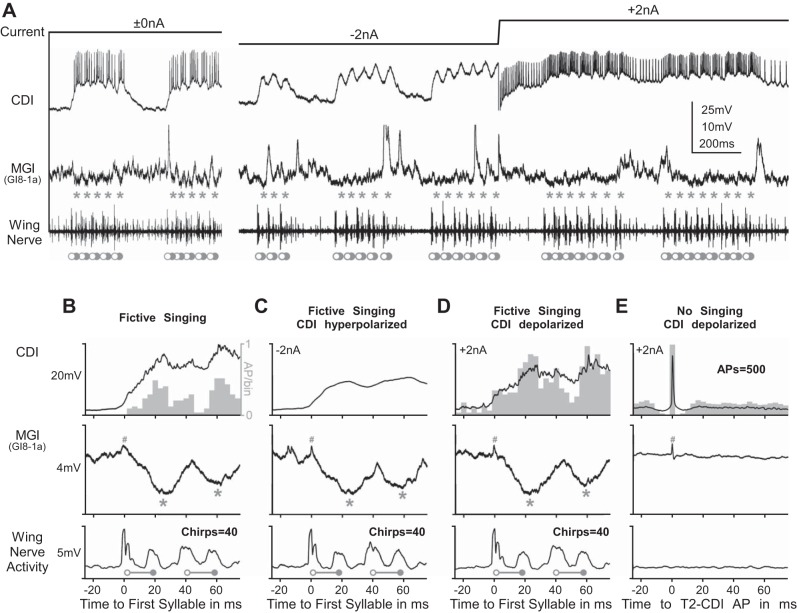
CDI of the median giant fiber (GI8-1a) during fictive singing is independent of CDI spike activity. *A*–*E*: intracellular recording and current injection in the CDI dendrite (*top* traces) and simultaneous intracellular recording of the GI8-1a dendrite (*middle* trace; asterisks indicate timing of postsynaptic CDI, which is often superimposed by a high background activity of spontaneous synaptic input and, therefore, may be masked in the original recording traces, but was always evident in signal averages). Open and solid circles indicate the opener and closer motoneuron activity in the wing nerve (*bottom* traces). *A*: vertical scaling bar is 25 mV for CDI and 10 mV for MGI; note that the MGI spikes have been truncated. *B*–*D*: quantitative data analysis with the histogram of CDI spike activity (gray bars) and the averaged recording signals (black lines) shows that decreasing (*C*) or increasing (*D*) the CDI spike activity by intracellular current injection did not change the amplitude or timing of CDI (gray asterisks) in the MGI dendrite. *E*: spike activity of the CDI driven by current injection in a resting animal does not induce postsynaptic inhibition in the MGI dendrite. #Artifact due to signal cross talk from trigger channel (*B*–*D*: triggered to first opener motoneuron spike of the chirp; *E*: triggered to CDI spikes).

A main candidate to mediate the corollary discharge to the cercal wind-sensitive system could be the identified mesothoracic CDI that modulates the activity in the auditory pathway during singing (Poulet and Hedwig 2006). This interneuron is rhythmically activated in phase with the singing motor pattern and its descending axon projects to the terminal ganglion. By simultaneous recordings from the dendrite of the CDI in the mesothoracic ganglion and the dendrite of the MGI in the terminal ganglion, we tested if the CDI also inhibits this wind-sensitive giant fiber during singing (Fig. 5*A*).

During each fictive chirp, the CDI depolarized and spiked in phase with the wing-closer motoneuron activity and the maximum of inhibition in the MGI (Fig. 5, *B*–*D*). When we modulated the spiking activity of the CDI by de- and hyperpolarizing current injection of 2 nA (Fig. 5*A*), neither increasing nor abolishing the CDI spike activity affected the amplitude or timing of the corollary discharge inhibition in the dendrite of the MGI (Fig. 5, *B*–*D*). After the cricket had stopped singing, driving tonic spike activity in the CDI to about 50 action potentials per second by 2 nA depolarizing current injection caused no change in the membrane potential of the MGI dendrite (Fig. 5*E*). Moreover, the onset of CDI depolarization during fictive singing, as well as the start of inhibition in the MGI (and also in GI8-1b), coincided strictly with the opener motoneuron burst in the wing nerve (Figs. 3*B*, 4*B*, 5, *B*–*D*). Considering further that CDI action potentials were recorded near the spike-generating zone in the mesothoracic ganglion and need about 10- to 15-ms conduction time from there to arrive in the terminal ganglion (i.e., 20 mm at 1.6 ms^−1^; cf., Poulet and Hedwig 2006), the timing of CDI spikes appears to be by far too late to be the source of the corollary discharge inhibition in the dendrites of GI8-1a (MGI) and GI8-1b.

**Fig. 6.**
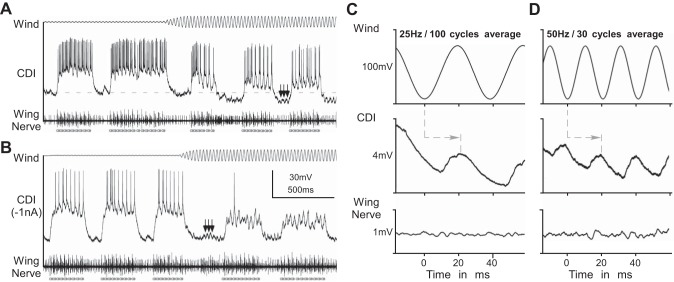
Cercal wind stimulation reduces CDI spike activity during fictive singing by postsynaptic inhibition. *A*–*D*: cercal wind stimulation (*top* traces) and intracellular recording of the CDI (*middle* trace) during fictive singing (*bottom* traces). Open and solid circles indicate the opener and closer motoneuron activity, respectively, in the wing nerve. *A* and *B*: singing-related spike activity of the CDI is reduced during 25-Hz sinusoidal wind stimulation. Wind stimulation-induced inhibition of the CDI is also indicated by the overall reduction (see dashed line in *A*) and 25-Hz oscillation (black arrows) of the dendritic membrane potential during the chirp intervals. *C* and *D*: quantitative data analysis shows the averaged membrane potential (black lines) for 25 Hz (*C*: *n* = 100; *N* = 1) and 50 Hz (*D*: *n* = 30; *N* = 1) wind stimulation. Rhythmic inhibition of the CDI is strictly time coupled to the wind stimulation (dashed lines with arrows indicate the constant delay to the start of inhibition).

In the example shown in Fig. 6, repetitive cercal wind stimulation significantly reduced the rhythmic spike activity of the CDI during fictive singing from an average of 4.7 to 2.1 action potentials per syllable (Fig. 6*A*). When the spike activity of the CDI was additionally reduced to 1–2 spikes per syllable by 1-nA hyperpolarizing current injection, the cercal wind stimulation abolished the remaining spiking activity of the CDI almost completely (Fig. 6*B*). During the chirp intervals of fictive singing, the membrane potential of the CDI showed periodic inhibitions (arrows in Fig. 6, *A* and *B*) occurring independent of the singing motor activity, but strictly in phase with the sinusoidal cercal wind stimulation (Fig. 6, *C* and *D*). Each wind stimulation cycle elicited inhibition in the CDI dendrite with a constant time delay of about 20 ms (25-Hz stimulation: Fig. 6*C*; 50-Hz stimulation: Fig. 6*D*; arrows indicate start of inhibition). Similar rhythmic inhibition of the CDI had been described by Poulet and Hedwig (2006) when they used wind stimulation to terminate singing and induce flight behavior in the cricket. Our data add to this earlier finding by showing that also during fictive singing the CDI receives inhibition that is time coupled to cercal wind stimulation.

## DISCUSSION

Corollary discharges are a widespread neural mechanism which link motor pattern generation with sensory processing (Sperry 1950; von Holst and Mittelstaedt 1950). They are considered to cancel or modulate self-generated sensory signals to allow better detection of simultaneously occurring external stimuli, protect sensory pathways from desensitization and prevent reafferent stimulation that leads to unnecessary reflexes (Crapse and Sommer 2008; Poulet and Hedwig 2002; Requarth and Sawtell 2014; Reznik et al. 2014). Here we investigated corollary discharge inhibition in the wind-sensitive cercal escape pathway in singing field crickets, one of the few established model systems where corollary discharge processing can be studied at the level of identified neurons (Poulet and Hedwig 2007).

### 

#### Corollary discharge modulation of cercal giant fibers.

In field crickets, the primary function of the cercal giant fiber pathway is to trigger fast escape reactions in response to air currents (Hörner 1992; Tauber and Camhi 1995) that indicate approaching predators like spiders (Dangles et al. 2006), digger wasps (Gnatzy 2001) and insectivore mammals (Langley 1981). During singing, a male cricket generates periodic airflow around its body due to the rhythmic wing movements underlying sound production (Kämper and Dambach 1985). These air currents are also detected by the crickets' cercal wind-sensitive afferents and elicit spiking in some ascending cercal interneurons (Kämper and Dambach 1981). Singing crickets, however, tolerate their self-generated airflow and also gentle cercal stimulation with short air puffs during the chirp phase, but they alter their chirping pattern, stop singing or even show escape reactions upon cercal stimulation with air puffs during chirp intervals (Dambach et al. 1983; Hedwig 2000; Lewkiewicz and Zuk 2004). The activity of those cercal interneurons, which elicit silencing and escape responses, must, therefore, be modulated or canceled during the chirp phase.

Our intracellular recordings demonstrate that the two prominent ventral GIs, GI8-1a (MGI) and GI8-1b, of the cercal escape pathway are rhythmically inhibited during fictive singing. In both interneurons, corollary discharge inhibition occurred strictly in phase with the motoneuron activity driving the wing-opener muscles for singing and significantly reduced their response to cercal wind stimuli during the fictive chirps. This rhythmic inhibition reduces giant fiber responses to self-generated air currents in sonorously singing cricket and hence prevents inadvertent silencing reactions or escape responses. The timing of the inhibition is in accord with the behavioral responses of singing crickets to cercal wind stimulation, as air puffs presented during the chirps had no effect (Dambach et al. 1983). Extracellular connective recordings demonstrated that some nonidentified ascending interneurons are phasically activated during sonorous singing (Kämper and Dambach 1981), but these multiunit recordings were not suitable to reveal corollary discharge inhibition in individual GIs of the cercal pathway. The situation may be similar to cricket walking behavior, where the spike activity of the ventral GIs is reduced, whereas the activity in the dorsal GIs is increased (Daley and Delcomyn 1980; Kohstall-Schnell and Gras 1994). This would also correspond to studies in cockroaches, which demonstrated a reduced response of ventral GIs to cercal wind stimulation during flight, whereas rhythmic responses of dorsal GIs contribute to the control of the wing-beat frequency. As the spike activity of ventral GIs can inhibit the flight motor pattern generation, a reduction of their responsiveness prevents reafferent feedback from interrupting the flight behavior (Libersat 1992).

#### Comparison of singing related corollary discharge inhibition in the wind-sensitive and auditory pathway.

Previous studies demonstrated that singing crickets protect their auditory pathway from self-induced desensitization by a corollary discharge mechanism (review: Poulet 2005). Presynaptic inhibition targets the synaptic transmission between auditory afferents and auditory interneurons, and postsynaptic inhibition impinges directly on first-order auditory interneurons (Poulet and Hedwig 2003). In the auditory pathway, the corollary discharge inhibition is precisely timed to the sound pulse generation to efficiently control the neuronal responses to the reafferent feedback (Poulet and Hedwig 2002). Similar to the first-order sensory interneurons in the auditory pathway, the ventral giant fibers GI8-1a and GI8-1b of the wind-sensitive pathway also receive precisely timed postsynaptic inhibition by rhythmic corollary discharge from the singing motor pattern. Our intracellular recordings from wind-sensitive cercal afferents, however, revealed no indication for primary afferent depolarization occurring in phase with the singing rhythm, which would reflect presynaptic inhibition at the first synapse of the wind-sensitive giant fiber pathway of the cricket. In contrast to the auditory pathway, where about 50 primary afferents connect to only 6 spiking interneurons (Wohlers and Huber 1982), the comparatively large number of wind-sensitive interneurons in the terminal ganglion indicates that sensory processing is much more complex and multifunctional in the cercal system (Baba et al. 1995; Bodnar et al. 1991; Boyan and Ball 1990; Jacobs and Murphey 1987; Jacobs and Theunissen 2000; Kobashi and Yamaguchi 1984; Kohstall-Schnell and Gras 1994). To prevent the wind-sensitive pathway from triggering of escape responses during singing, it is sufficient to target only the dendrites of specific GIs by corollary discharge inhibition, which would still allow processing of the “unfiltered” sensory inputs by other parts of the cercal wind-sensitive pathway (e.g., local and ascending non-GIs).

Besides central control by corollary discharge (Murphey and Palka 1974; Delcomyn 1977; Blagburn and Sattelle 1987; Levine and Murphey 1980), other ways of controlling self-generated sensory feedback have been found in the cercal system of different insects. Some katydids seem to avoid the self-stimulation of the wind-sensitive sensilla during singing by shielding the cerci with their folded hind wings and by a singing posture that increases the distance between the moving front wings and the cerci (Hartbauer et al. 2010). In flying cockroaches, the flow of afferent cercal activity is reduced by a mechanical block of the cercal nerve (Libersat and Camhi 1988). In the locust, an afferent unit at the base of each cercus detects movement of the cerci; it causes primary afferent depolarizations reflecting presynaptic inhibition in the axon terminals of filiform hairs (Boyan 1988) and, consequently, desensitizes the cercal pathway for the time of self-induced afferent input.

#### Neuronal substrate of corollary discharge during singing.

Rhythmic inhibition of auditory afferents and first-order auditory interneurons is mediated by the same identified CDI, which is activated strictly in phase with the closer activity of the singing motor network (Poulet and Hedwig 2006). The morphology of the CDI with axonal projections in all ganglia of the cricket's central nervous system, including the TAG, suggests that this neuron can simultaneously modulate other sensory pathways as well. Our recordings, however, show no evidence to support a corollary discharge function of this CDI in the cercal pathway, where the recorded interneurons were already inhibited in the opener phase of the singing motor activity, i.e., before the production of sound pulses by wing-closer activity. Simultaneous recordings of the MGI (GI8-1a) and the CDI did not show any evidence for synaptic input from the CDI to the MGI. Driving spike activity in the CDI by depolarizing current injection also had no effect on the dendritic membrane potential of the MGI. Furthermore, CDI spikes need to travel from the mesothoracic ganglion to the TAG. Due to this long spatial distance, the timing of CDI spike activity in the wing-closer phase occurs too late to cause the inhibition of the cercal GIs in the wing-opener phase. We, therefore, conclude that, in parallel to the previously identified CDI, another corollary discharge pathway must exist that drives the inhibition in ventral GIs of the cercal pathway. Until further experimental evidence is provided, the function of the axonal branches of the CDI in the terminal ganglion, as well as the neuronal pathway providing the corollary discharge to the ventral GIs, remains open for speculation. As the singing network extends into the abdominal ganglia (Schöneich and Hedwig 2011), descending opener-interneurons of the singing central pattern generator (like the T3-DO interneuron; Schöneich and Hedwig 2012) could directly mediate a corollary discharge of the motor pattern to the cercal system. A descending inhibitory corollary discharge from thoracic ganglia to giant fibers in the terminal ganglion has been demonstrated in walking crickets (Murphey and Palka 1974) and suggested in walking cockroaches (Delcomyn 1977).

#### Modulation of corollary discharge by sensory input.

To adjust the timing of corollary discharges to the self-stimulation of the different sensory pathways, a singing cricket cannot rely solely on the CDI previously identified by Poulet and Hedwig (2006). The cricket central nervous system rather seems to employ a distributed network organization of at least two parallel corollary discharge pathways to adequately target different sensory systems in a time-specific manner. Such an organization of corollary discharges may be comparable to processing in active sensing animals such as weakly electric fish and echolocating bats, where different sensory systems are targeted in parallel by corollary discharge mechanisms at the same time (Crapse and Sommer 2008). However, this might not be the only reason for the parallel corollary discharge pathways. Our experiments demonstrate that the CDI, mediating the modulation of the auditory response, is inhibited upon cercal wind stimulation via a pathway ascending from the terminal ganglion. As the CDI interneuron was previously shown to be inhibited during flight behavior elicited by air blown toward the animal (Poulet and Hedwig 2006), we propose that at least part of this inhibition may have resulted from activating the cercal pathway by the airflow. Inhibition of the CDI upon external cercal stimulation will reduce its inhibitory impact on the auditory pathway during singing and, consequently, increase the responsiveness to acoustic signals whenever the cercal pathway detects an approaching predator or another potential threat. A similar inhibitory connection may also exist between the auditory pathway and the yet unidentified CDI that inhibits the wind-sensitive ventral GIs during singing. Although there is no direct evidence for the behavioral relevance of such cross-modal corollary discharge suppression yet, we speculate that it may allow for a more flexible and situation-specific adjustment of response thresholds in the sensory pathways during the singing behavior.

## GRANTS

This study was supported by the Biotechnology and Biological Science Research Council (Grant BB/F008783/1) and The Isaac Newton Trust (Trinity College, Cambridge, UK).

## DISCLOSURES

No conflicts of interest, financial or otherwise, are declared by the author(s).

## AUTHOR CONTRIBUTIONS

Author contributions: B.H. and S.S. conception and design of research; S.S. performed experiments; S.S. analyzed data; S.S. and B.H. interpreted results of experiments; S.S. prepared figures; S.S. drafted manuscript; S.S. and B.H. edited and revised manuscript; S.S. and B.H. approved final version of manuscript.

## References

[B1] Baba Y, Hirota K, Shimozawa T, Yamaguchi T Differing afferent connections of spiking and nonspiking wind-sensitive local interneurons in the terminal abdominal ganglion of the cricket *Gryllus bimaculatus*. J Comp Physiol A 176: 17–30, 1995.

[B2] Bell CC Sensory coding and corollary discharge effects in mormyrid electric fish. J Exp Biol 146: 229–53, 1989. 2689564 10.1242/jeb.146.1.229

[B3] Blagburn JM, Sattelle DB Presynaptic depolarization mediates presynaptic inhibition at a synapse between an identified mechanosensory neurone and giant interneurone 3 in the first instar cockroach, *Periplaneta americana*. J Exp Biol 127: 135–157, 1987.

[B4] Bodnar DA, Miller JP, Jacobs GA Anatomy and physiology of identified wind-sensitive local interneurons in the cricket cercal sensory system. J Comp Physiol A 168: 553–564, 1991. 1920156 10.1007/BF00215077

[B5] Boyan GS Presynaptic inhibition of identified wind-sensitive afferents in the cercal system of the locust. J Neurosci 8: 2748–2757, 1988. 3411352 10.1523/JNEUROSCI.08-08-02748.1988 PMC6569393

[B6] Boyan GS, Ball EE Neuronal organization and information processing in the wind-sensitive cercal receptor/giant interneurone system of the locust and other orthopteroid insects. Prog Neurobiol 35: 217–243, 1990. 2236578 10.1016/0301-0082(90)90028-f

[B7] Crapse TB, Sommer MA Corollary discharge across the animal kingdom. Nat Rev Neurosci 9: 587–600, 2008. 18641666 10.1038/nrn2457 PMC5153363

[B8] Cullen KE Sensory signals during active versus passive movement. Curr Opin Neurobiol 14: 698–706, 2004. 15582371 10.1016/j.conb.2004.10.002

[B9] Daley DL, Delcomyn F Modulation of the excitability of cockroach giant interneurons during walking. I. Simultaneous excitation and inhibition. J Comp Physiol A 138: 231–239, 1980.

[B10] Dambach M, Rausche HG, Wendler G Proprioceptive feedback influences the calling song of the field cricket. Naturwissenschaften 70: 417–418, 1983.

[B11] Dangles O, Ory N, Steinmann T, Christides JP, Casas J Spider's attack versus cricket's escape: velocity modes determine success. Anim Behav 72: 603–610, 2006.

[B12] Davis WJ, Siegler MVS, Mpitsos GJ Distributed neuronal oscillators and efference copy in the feeding system of pleurobranchaea. J Neurophysiol 36: 258–274, 1973. 4350359 10.1152/jn.1973.36.2.258

[B13] Delcomyn F Corollary discharge to cockroach giant interneurones. Nature 269: 160–162, 1977. 909581 10.1038/269160a0

[B14] Feinberg I, Guazzelli M Schizophrenia–a disorder of the corollary discharge systems that integrate the motor systems of thought with the sensory systems of consciousness. Br J Psychiatry 174: 196–204, 1999. 10448443 10.1192/bjp.174.3.196

[B15] Ferreira M, Ferguson JWH Geographic variation in the calling song of the field cricket *Gryllus bimaculatus* (*Orthoptera*: *Gryllidae*) and its relevance to mate recognition and mate choice. J Zool Lond 257: 163–170, 2002.

[B16] Gnatzy W Digger wasp vs. cricket: (neuro-) biology of a predator-prey-interaction. Zoology 103: 125–139, 2001.

[B17] Gnatzy W, Hustert R Mechanoreceptors in behavior. In: Cricket Behavior and Neurobiology, edited by Huber F, Moore TE, Loher W Ithaca, NY: Cornel University Press, 1989, p. 198–226.

[B18] Gras H, Hörner M Wind evoked running of the cricket *Gryllus bimaculatus*. I. Behavioural analysis. J Exp Biol 171: 189–214, 1992.

[B19] Hartbauer M, Ofner E, Grossauer V, Siemers BM The cercal organ may provide singing Tettigoniids a backup sensory system for the detection of eavesdropping bats. PLos One 5: e12698, 2010. 20856887 10.1371/journal.pone.0012698 PMC2938355

[B20] Hedwig B Control of cricket stridulation by a command neuron: efficacy depends on the behavioral state. J Neurophysiol 83: 712–722, 2000. 10669487 10.1152/jn.2000.83.2.712

[B21] Hörner M Wind-evoked escape running of the cricket *Gryllus bimaculatus*. II. Neurophysiological analysis. J Exp Biol 171: 215–245, 1992.

[B22] Jacobs GA, Murphey RK Segmental origins of the cricket giant interneuron system. J Comp Neurol 265: 145–157, 1987. 3693602 10.1002/cne.902650110

[B23] Jacobs GA, Theunissen FE Extraction of sensory parameters from a neural map by primary sensory interneurons. J Neurosci 20: 2934–2943, 2000. 10751446 10.1523/JNEUROSCI.20-08-02934.2000 PMC6772197

[B24] Kämper G, Dambach M Low-frequency airborne vibrations generated by crickets *Gryllus bimaculatus* during singing and aggression. J Insect Physiol 31: 925–930, 1985.

[B25] Kämper G, Dambach M Response of the cercus-to-giant interneurone system in crickets to species-specific song. J Comp Physiol A 141: 311–317, 1981.

[B26] Kanou M, Ohshima M, Inoue J The air-puff evoked escape behavior of the cricket *Gryllus bimaculatus* and its compensational recovery after cercal ablations. Zool Sci 16: 71–79, 1999.

[B27] Knepper M, Hedwig B NEUROLAB, a PC-program for the processing of neurobiological data. Comput Methods Programs Biomed 52: 75–77, 1997. 9034672 10.1016/s0169-2607(96)01781-6

[B28] Kobashi M, Yamaguchi T Local non-spiking interneurons in the cercus-to-giant interneuron system of crickets. Naturwissenschaften 71: 154–156, 1984.

[B29] Kohstall-Schnell D, Gras H Activity of giant interneurones and other wind-sensitive elements of the terminal ganglion in the walking cricket. J Exp Biol 193: 157–181, 1994. 9317546 10.1242/jeb.193.1.157

[B30] Kumagai T, Shimozawa T, Baba Y The shape of wind-receptor hairs of cricket and cockroach. J Comp Physiol A 183: 187–192, 1998.

[B31] Landolfa MA, Miller JP Stimulus-response properties of cricket cercal filiform receptors. J Comp Physiol A 177: 749–757, 1995.

[B32] Langley W The effect of prey defenses on the attack behavior of the southern grasshopper mouse (*Onychomys torridus*). Z Tierpsychol 56: 115–127, 1981.

[B33] Levine RB, Murphey RK Pre- and postsynaptic inhibition of identified giant interneurons in the cricket (*Acheta domesticus*). J Comp Physiol A 135: 269–282, 1980.

[B34] Levy RA The role of GABA in primary afferent depolarization. Prog Neurobiol 9: 211–267, 1977. 205909 10.1016/0301-0082(77)90002-8

[B35] Lewkiewicz DA, Zuk M Latency to resume calling after disturbance in the field cricket, *Teleogryllus oceanicus*, corresponds to population-level differences in parasitism risk. Behav Ecol Sociobiol 55: 569–573, 2004.

[B36] Libersat F Modulation of flight by the giant interneurons of the cockroach. J Comp Physiol A 170: 379–392, 1992.

[B37] Libersat F, Camhi JM Control of cercal position during flight in the cockroach: a mechanism for regulating sensory feedback. J Exp Biol 136: 483–488, 1988. 3404078 10.1242/jeb.136.1.483

[B38] Magal C, Dangles O, Caparroy P, Casas J Hair canopy of cricket sensory system tuned to predator signals. J Theor Biol 241: 459–466, 2006. 16427653 10.1016/j.jtbi.2005.12.009

[B39] Matsumoto SG, Murphey RK The cercus-to-giant interneuron system of crickets. IV. Patterns of connectivity between receptors and the medial giant interneuron. J Comp Physiol A 119: 319–330, 1977.

[B40] Meyer J, Hedwig B The influence of tracheal pressure changes on the responses of the tympanal membrane and auditory receptors in the locust *Locusta migratoria*. J Exp Biol 198: 1327–1339, 1995. 9319210 10.1242/jeb.198.6.1327

[B41] Miller JP, Krueger S, Heys JJ, Gedeon T Quantitative characterization of the filiform mechanosensory hair array on the cricket cercus. PLos One 6: e27873, 2011. 22132155 10.1371/journal.pone.0027873 PMC3221685

[B42] Murphey RK, Palka J Efferent control of cricket giant fibres. Nature 248: 249–251, 1974.

[B43] Nishi S, Minota S, Karczmar AG Primary afferent neurons: the ionic mechanism of GABA-mediated depolarization. Neuropharmacology 13: 215–219, 1974. 4546218 10.1016/0028-3908(74)90110-5

[B44] Pollack GS, Givois V, Balakrishnan R Air-movement “signals” are not required for female mounting during courtship in the cricket *Teleogryllus oceanicus*. J Comp Physiol A 183: 513–518, 1998.

[B45] Poulet JFA Corollary discharge inhibition and audition in the stridulating cricket. J Comp Physiol A 191: 979–986, 2005. 10.1007/s00359-005-0027-z 16249882

[B46] Poulet JFA, Hedwig B A corollary discharge maintains auditory sensitivity during sound production. Nature 418: 872–876, 2002. 12192409 10.1038/nature00919

[B47] Poulet JFA, Hedwig B A corollary discharge mechanism modulates central auditory processing in singing crickets. J Neurophysiol 89: 1528–1540, 2003. 12626626 10.1152/jn.0846.2002

[B48] Poulet JFA, Hedwig B New insights into corollary discharges mediated by identified neural pathways. Trends Neurosci 30: 14–21, 2007. 17137642 10.1016/j.tins.2006.11.005

[B49] Poulet JFA, Hedwig B The cellular basis of a corollary discharge. Science 311: 518–522, 2006. 16439660 10.1126/science.1120847

[B50] Pynn LK, DeSouza JFX The function of efference copy signals: implications for symptoms of schizophrenia. Vision Res 76: 124–133, 2013. 23159418 10.1016/j.visres.2012.10.019

[B51] Requarth T, Sawtell NB Plastic corollary discharge predicts sensory consequences of movements in a cerebellum-like circuit. Neuron 82: 896–907, 2014. 24853945 10.1016/j.neuron.2014.03.025 PMC4032477

[B52] Reznik D, Henkin Y, Schadel N, Mukamel R Lateralized enhancement of auditory cortex activity and increased sensitivity to self-generated sounds. Nat Commun 5: 4059, 2014. 24898564 10.1038/ncomms5059

[B53] Robertson RM, Moulins M A corollary discharge of total foregut motor activity is monitored by a single interneurone in the lobster *Homarus gammarus*. J Physiol 77: 823–827, 1981. 7341761

[B54] Schöneich S, Hedwig B Cellular basis for central motor pattern generation in singing crickets. Brain Behav 2: 707–725, 2012. 23170234 10.1002/brb3.89 PMC3500458

[B55] Schöneich S, Hedwig B Hyperacute directional hearing and phonotactic steering in the cricket (*Gryllus bimaculatus* deGeer). PLos One 5: e15141, 2010. 21170344 10.1371/journal.pone.0015141 PMC2999563

[B56] Schöneich S, Hedwig B Neural basis of singing in crickets: central pattern generation in abdominal ganglia. Naturwissenschaften 98: 1069–1073, 2011. 22038326 10.1007/s00114-011-0857-1

[B57] Schöneich S, Schildberger K, Stevenson PA Neuronal organization of a fast-mediating cephalothoracic pathway for antennal-tactile information in the cricket (*Gryllus bimaculatus* DeGeer). J Comp Neurol 519: 1677–1690, 2011. 21452239 10.1002/cne.22594

[B58] Shepherd D, Kämper G, Murphey RK The synaptic origins of receptive field properties in the cricket cercal sensory system. J Comp Physiol A 162: 1–11, 1988.

[B59] Shimozawa T, Kanou M Varieties of filiform hairs: range fractionation by sensory afferents and cercal interneurons of a cricket. J Comp Physiol A 155: 485–493, 1984.

[B60] Sperry RW Neural basis of the spontaneous optokinetic response produced by visual inversion. J Comp Physiol Psychol 43: 482–489, 1950. 14794830 10.1037/h0055479

[B61] Tauber E, Camhi JM The wind-evoked escape behavior of the cricket Gryllus *bimaculatus*: integration of behavioral elements. J Exp Biol 198: 1895–1907, 1995. 9319804 10.1242/jeb.198.9.1895

[B62] Tobias M, Murphey RK The response of cercal receptors and identified interneurons in the cricket (*Acheta domesticus*) to airstreams. J Comp Physiol A 129: 51–59, 1979.

[B63] von Holst E, Mittelstaedt H Das Reafferenzprinzip: Wechselwirkungen zwischen Zentralnervensystem und Peripherie. Naturwissenschaften 37: 464–476, 1950.

[B64] Wenzel B, Elsner N, Hedwig B Microinjection of neuroactive substances into brain neuropile controls stridulation in the cricket *Gryllus bimaculatus* (de Geer). Naturwissenschaften 85: 452–454, 1998.

[B65] Wohlers DW, Huber F Processing of sound signals by six types of neurons in the prothoracic ganglion of the cricket, *Gryllus campestris* L. J Comp Physiol A 146: 161–173, 1982.

[B66] Yang Y, Cao P, Yang Y, Wang SR Corollary discharge circuits for saccadic modulation of the pigeon visual system. Nat Neurosci 11: 595–602, 2008. 18391942 10.1038/nn.2107

[B67] Zaretsky M, Rowell CH Saccadic suppression by corollary discharge in the locust. Nature 280: 583–585, 1979. 460439 10.1038/280583a0

